# Understanding and treating diabetic foot ulcers: Insights into the role of cutaneous microbiota and innovative therapies

**DOI:** 10.1002/ski2.399

**Published:** 2024-05-30

**Authors:** Paul Norton, Pavlos Trus, Fengyi Wang, M. Julie Thornton, Chien‐Yi Chang

**Affiliations:** ^1^ School of Dental Sciences Faculty of Medical Sciences Newcastle University Newcastle Upon Tyne UK; ^2^ Biosciences Institute Faculty of Medical Sciences Newcastle University Newcastle Upon Tyne UK; ^3^ Centre for Skin Sciences Faculty of Life Sciences University of Bradford Bradford UK

## Abstract

**Background:**

Notoriously known as the silent pandemic, chronic, non‐healing diabetic foot ulcers (DFUs), pose a significant rate of incidence for amputation and are a major cause of morbidity. Alarmingly, the treatment and management strategies of chronic wounds represent a significant economic and health burden as well as a momentous drain on resources with billions per annum being spent in the US and UK alone. Defective wound healing is a major pathophysiological condition which propagates an acute wound to a chronic wound, further propelled by underlying conditions such as diabetes and vascular complications which are more prevalent amongst the elderly. Chronic wounds are prone to infection, which can exacerbate the condition, occasionally resulting in amputation for the patient, despite the intervention of modern therapies. However, amputation can only yield a 5‐year survival rate for 50% of patients, highlighting the need for new treatments for chronic wounds.

**Findings:**

The dynamic cutaneous microbiota is comprised of diverse microorganisms that often aid wound healing. Conversely, the chronic wound microbiome consists of a combination of common skin commensals such as *Staphylococcus aureus* and *Staphylococcus epidermidis*, as well as the opportunistic pathogen *Pseudomonas aeruginosa*. These bacteria have been identified as the most prevalent bacterial pathogens isolated from chronic wounds and contribute to prolific biofilm formation decreasing the efficiency of antimicrobials and further perpetuating a hyper‐inflammatory state.

**Discussion and Conclusion:**

Here, we review recent advances and provide a new perspective on alternative treatments including phage and microbiome transplant therapies and how the definitive role of the cutaneous microbiota impacts the aetiology of DFUs.

## Graphical abstract

The silent pandemic, chronic, non‐healing diabetic foot ulcers (DFUs), pose a significant rate of incidence for amputation and are a major cause of morbidity. The dynamic cutaneous microbiota is comprised of diverse microorganisms that often play a sword with double edged by causing opportunistic infections or aiding wound healing. We review recent advances in wound healing and provide new perspective on alternative treatments including phage and microbiome transplant therapies and how the definitive role of the cutaneous microbiota impacts the aetiology of DFUs.

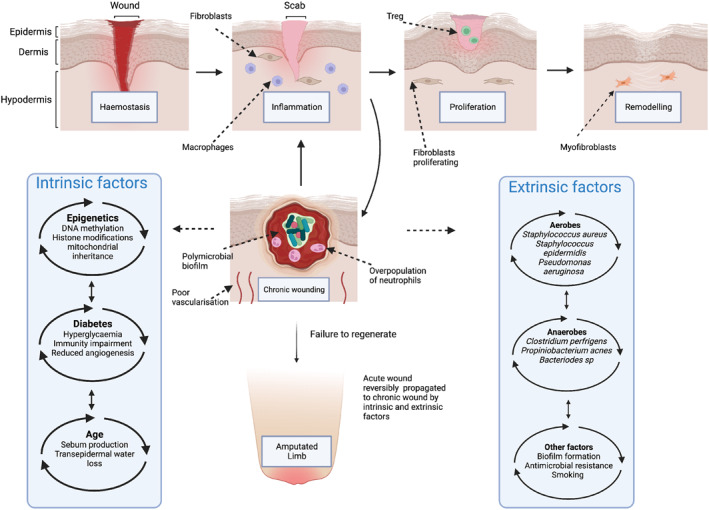

**What is already known about this topic?**

- Chronic, non‐healing diabetic foot ulcers (DFUs) and associated infections are a significant health issue, often leading to amputation and causing substantial morbidity.
- The cutaneous microbiota, comprising diverse microorganisms, plays a crucial role in wound healing.

**What does this study add?**

- The purpose of this article is to highlight the profound impact of microbes in wound infections and healing, and to provide new perspective on new treatment strategy.

## 1 INTRODUCTION

The skin is a complex, intricate tissue responsible for maintaining fundamental standard homoeostatic controls which regulate our external and internal physiology.^1^ The structure, along with its appendages, including hair follicles, are paramount in providing an essential obstacle to opportunistic pathogens, as well as, maintaining an anatomical barrier which protects intrinsic architecture from physical, chemical, and biological impairment.^2^ For mammals, the skin is chiefly branched into three core layers namely the epidermis, dermis and hypodermis or subcutaneous tissue. The outermost layer of our skin, epidermis, is covered in microorganisms with their composition across skin sites differing drastically. Locations which contain more sebaceous glands usually contain the greatest bacterial load mainly dominated by staphylococci and *Propionibacterium* species. Warmer sites contain larger amounts of staphylococci and *Corynebacterium*, while locations that are usually dry contain the greatest diversity of microorganisms with a higher number of *Flavobacterium* and *β‐Proteobacteria*.^3^, ^4^ Research has shown that the microbiota extends its reach into sub‐epidermal appendages including hair follicles and sebaceous glands with larger abundancies of *Actinobacteria* (*Corynebacterium*) and *Proteobacteria* (*Acinetobacter)* and lower proportions of Firmicutes (*Bacilli*, *Lactobacillus*).^5^ It highlights the diversity of bacteria and eliminates previous assumptions about the role of the microbiota's capacity to colonise deep into the dermis.

**FIGURE 1:**
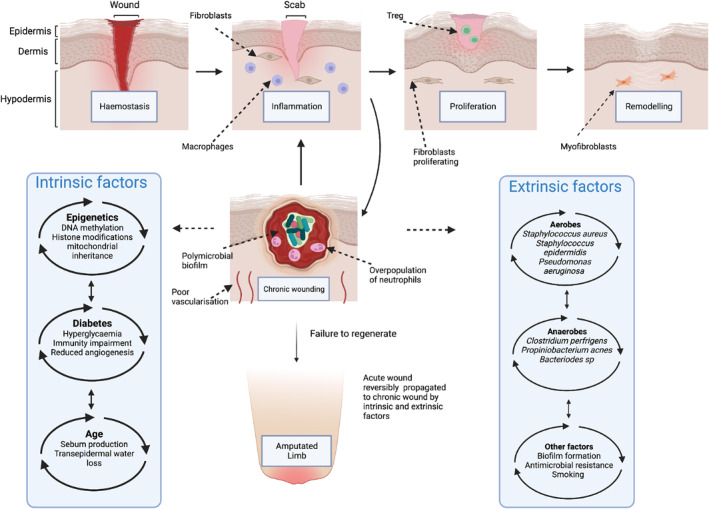
Schematic of extrinsic and intrinsic aberrations in chronic wounding. An acute wound usually follows the linear paradigm of routine healing following, haemostasis, inflammation, proliferation, and remodelling. However extrinsic (aerobes, anaerobes) and intrinsic factors (epigenetics, diabetes, age) can stall the wound at the inflammatory stages of wounding, hyper‐perpetuating the inflammatory response, leading to chronic wounding and in some cases, amputation, and death.

When the skin is subject to a physical insult such as a wound, repair mechanisms are engaged which governs the regenerative process.^3^ This process can be categorised by four intricate, sequential, and overlapping stages; haemostasis (coagulation), inflammation, proliferation and finally remodelling.^4^ These stages must function in unison to effectively re‐establish the barrier function of the skin to maintain homoeostasis, ultimately resulting in re‐epithelialisation and repair of underlying connective tissue. Wounds can be broadly classified into two types: acute and chronic. Most acute wounds will heal and traverse all four stages of healing within two to 4 weeks, the ‘normal’ and ‘healthy’ process of regeneration.^6^ Wounds that stall within any stages of the regeneration cascade and do not heal within 3 months are classified as chronic. Chronic wounds can arise when there is a failure to regenerate and they become stalled usually in the initial inflammatory stage of wound healing, despite all modern therapeutics and therapies available (Figure 1).^7^ This often occurs because of an underlying disease, such as diabetes or immunocompromised state.

Chronic wounds associated with diabetic foot ulcers (DFUs), venous leg ulcers (VLUs) and pressure sores are a major burden on healthcare and economy. It is estimated the expenditure spent on treating patients with chronic wounds in the year of 2022 in the UK is 8.83 billion GBP and in the US is 145 billion USD.^8^ With an ageing population, these figures are expected to rise.^9^ Chronic wounds are very difficult to treat, and patients usually need continuous therapeutic treatment including prolonged hospitalisation. Despite the advances in modern and personalised medicines, many chronic wounds degenerate further becoming non‐healing chronic wounds, that are further exacerbated by the presence of invading, opportunistic microorganisms.^10^ Infection can rapidly progress and invade into deeper tissues and increase the abundance of necrotic tissue resulting in amputation in 15%–27% of patients with chronic wounds. Amputation does increase life expectancy by up to 2 years, however it is estimated that only 56% of patients will survive longer than 5 years after the initial manifestation.^11^ These alarming figures highlight the need for new technologies and smart dressings which can effectively combat infections in chronic wounds.

## 2 THE DIABETIC FOOT AND DEFECTIVE HEALING

The routine paradigm of wound healing has been vital in the decipherment of the molecular, cellular, physiological and biochemical components which underpin the biology of tissue repair. However, even during the normal linear progression of wound healing, a wide range of factors have been known to influence aberrant repair including, thrombosis, ischaemia and infection. These aberrant repair systems develop as both environmental, genetic and epigenetic mechanisms that influence disease and contribute further to the pathophysiological abnormalities of chronic wounds.^12^ Experimental and clinical evidence suggests that DFUs do not follow the linear paradigm of cutaneous repair.^13^, ^14^ This is a result of a multitude of intrinsic and extrinsic factors including vascular complications, neuropathy, callus formation, nutrient deficiency, and hypoxia.^10^, ^15^ Predisposition to these abnormalities is referred to as the pathological triad of ischaemia, neuropathy and trauma.^16^, ^17^ Additionally, infection has its own subcategory which plays a vital role in chronic wound healing impairment. During the triad, one aberration leads to another resulting in a pathogenic cascade of vicious cycles in DFUs (Figure 1).^18^

DFUs are prevalent amongst the diabetic community, especially when diabetes is poorly managed resulting in hyperglycaemia. Hyperglycaemia alone leads to decreasing sensation in injuries and impairment of local immune responses.^19^ Moreover, the reduced microbiocidal activity of polymorphonuclear cells, which are more prevalent in the presence of hyperglycaemia, are a major contributing factor in the establishment of wound infections.^15^ Perhaps the largest contributing factor of a DFU is the altered angiogenic state resulting from the destruction of the vascular system and peripheral neuropathy with perturbations arising from diabetic hyperglycaemia.^20^ Inadequate angiogenesis also further hyper‐perpetuates the non‐healing state of the chronic wound which shows decreased capillary density and vascularity. Moreover, the anti‐angiogenic pigment epithelium derived factor has been reported to circulate at higher levels in patients with DFUs.^21^ This underlying delayed chronic wound state gives rise for the attachment and invasion of opportunistic pathogens. Not all chronic wounds start out infected, the neuropathy of delayed healing creates an enticing, nutrient rich environment for pathogens, usually with more than one bacterial species such as; *Staphylococcus*, *Pseudomonas* and *Stenotrophomonas*.^22^ These bacteria do not elicit a chronic wound, they hyper‐perpetuate the initial inflammatory stage of healing and become key players in maintain the wound in this state. Moreover, hyperglycaemic glucose‐rich diabetic abscess were found to significantly increase *Staphylococcus aureus* virulence potential^23^

Neuropathy initially constitutes a pain‐free condition for those who are subject to minor cutaneous breaches. Local paraesthesia on the plantar pressure points of the foot (hallux, heel and metatarsophalangeal joint) leads to formation of ulcers arising from minor physical insults which are improperly treated.^24^ Neuropathic ulcers typically affect sensory cutaneous nerves which are responsible for detecting pain and temperature.^25^ Therefore, those who suffer from diabetes and are subject to minor cuts on the plantar region of the foot, can remain unaware due to the maceration of the vascular system and neuropathy. These individuals fail to receive effective treatment leading to development of a non‐healing DFU due to aberrations in the vascular supply and angiogenic healing.^26^ Clearly there are underlying molecular, cellular, biochemical, and physiological mechanisms which contribute to this chronic wound state far before an infection establishes. As mentioned above, not all chronic wounds are initially infected, but the nutrient rich environment and change in immunity make the chronic wound microenvironment susceptible to invading opportunistic microorganisms that will proliferate and thrive. While infection is not the root cause of DFUs, microbes produce virulence factors and form aggregations or biofilms which hyper‐perpetuate the initial inflammatory state of healing and impair regeneration.^27^

Metabolic syndrome resulting in a proinflammatory state with the combination of obesity, insulin resistance, hypertension, and dyslipidaemia can significantly impact delayed healing.^28^ A link between obesity and wound healing has been unequivocally established, usually coupled with aberrant inflammation. Obesity can impact cutaneous sweat glands, sebaceous glands, barrier function, collagen organisation and effective wound healing with complications including anastomotic leaks, infection, and seromas.^29^ Vascular aberrations and reduced populations of immune cells prolong the initial inflammatory stage of wound healing. Macro‐ and micro‐nutrient deficiencies also play determining roles in impaired wound healing in obese individuals.^30^

There are several intrinsic factors that can delay the DFU healing process. Single nucleotide polymorphism can play a crucial role in the fluctuation of growth factors and cytokines which can impact DFU pathogenesis. Down regulation of fundamental growth factors and cytokines such as vascular endothelial growth factor, platelet derived growth factor, fibroblast growth factor and insulin like growth factor, has been identified as a key feature in the delayed healing of DFUs.^31^ A recent study^32^ has reported that increased expression of soluble Feline McDonough Sarcoma‐like tyrosine kinase‐1, correlated with oxidative stress, inflammation and angiogenesis, further perpetuates the progression of DFUs, while another showed elevated kallikrein binding protein impairs wound healing via induction of macrophage polarization.^33^ Epigenetic alterations for example, DNA methylation, histone modification, mitochondrial inheritance, and non‐coding RNAs, along with infection, can lead to the pathogenesis of diabetes and ultimately the development of a DFU.^34^, ^35^, ^36^, ^37^, ^38^

## 3 THE MICROBIOTA OF HEALTHY SKIN AND DFU CHRONIC WOUNDS

It is estimated that there are over 100 trillion microorganisms which take residence on, and within, the human body, far more than our own cells.^39^ A large amount of the human microbiota is found in the gut, where microorganisms are known to play pivotal roles in our well‐being.^40^ The microbiota is not constant, the composition differs significantly from person to person and many of our lifestyle choices make a substantial impact. The most impactful changes occur from early infancy for example, mode of birth, feeding, maternal nutrition status and intrapartum antibiotic prophylaxis, those impact the plasticity of the infant skin microbiota for maintaining homoeostasis and the delicate balance with the immune system.^41^ Throughout life, many factors can drastically impact and alter one's microbiota, including age, gender, diet, smoking status and geographical location.^42^

The skin microbiota is a diverse polymicrobial community that can influence the healing process of cutaneous wounds. The skin microbiota remains less understood than its gastrointestinal counterpart, however it is known to play a crucial role in the regulation of health and disease.^43^ Dysbiosis of the skin microbiota contributes to a wide array of atopic diseases including Job's syndrome, eczema, acne, vulgaris and psoriasis,^44^ usually as the result of the imbalance of one particular microorganism, *Cutibacterium acnes*.^45^ The microbiota can either help proliferate the establishment of an infection or promote a localised immune response that stimulates tissue repair. However, this depends on bacterial load, strain specificity and the severity of patient's underlying systemic disease. Over a thousand bacterial species and 19 phyla have been identified on the outermost layer of the skin. However, a select few phyla have been distinguished as the most common colonisers; *Actinobacteria*, *Firmicutes*, *Proteobacteria*, and *Bacteroidetes*, moreover the most represented genera include *Corynebacterium*, *Propionibacterium* and *Staphylococcus*.^46^ The virome and mycobiome are also important colonisers of the cutaneous microbiota. Species such as human papilloma virus, *Malassezia*, *Candida*, *Aspergillus* and *Cryptococcus* regulate the presence of bacterial species on the skin.^47^ Advances in polymerase chain reaction amplicon sequencing on broad spectrum 16S and 18S rDNA and large‐scale metagenomics projects have accurately identified the wide array of microorganisms present on the skin, however exact numbers are difficult to determine.^48^, ^49^, ^50^, ^51^, ^52^, ^53^, ^54^

Chronic wounds suffer from an overabundance of bacterial load. A load of 10^2^∼10^3^ colony forming units (CFU) can inflict laceration or abrasion trauma at the wound, whereas 10^6^ or more CFU impacts the normal physiological mechanics of healing.^55^, ^56^, ^57^ In the chronic wound, there are four states of infection. Firstly, there is colonisation of the wound bed from commensal microbes, which does not elicit an immune response. Secondly, the wound starts to become infected which is initiated by colonisation thereby forming micro colonies on the wound bed. Moreover, impaired immunity and antibiotic treatments can lead to over proliferation of resistant strains (*S*. *aureus* community acquired). The third stage consists of persistent critical colonisation or sub‐clinical infection and is characterised by a systemic delay in wound healing, but an inflammatory response has still not been elicited. This is thought to be caused by the virulence factors excreted by microorganisms to silence an immune response. This stage is the pivotal moment on whether the wound heals or not. Finally, microbial infection progresses to the final stage characterised by the proliferation of microbes, the invasion into viable tissues triggering an immune response, and eventually the formation of a polymicrobial biofilm for chronic infection. Acquisition of opportunistic nosocomial pathogens such as *P. aeruginosa* and *S*. *aureus* with an extensive antimicrobial profile can dominate the site of infection, ultimately leading to severe sepsis and death.^58^

Research has shown three main phyla identified in DFUs are similar to healthy commensals (*Actinobacteria*, *Firmicutes*, and *Proteobacteria*) found on ‘healthy skin’ highlighting the somewhat microbiological diversity of a DFU. However, it is important to note that most chronic wounds present with vastly different neuropathic and microbiological conditions, with each wound presenting with different shapes, sizes and underlying issues which impacts the colonisation of healthy or pathogenic microbes.^59^ Moreover, the chronic wound microbiome can house a polymicrobial community of healthy and pathogenic bacteria. It is the presence of these pathogenic microorganisms that can further perpetuate a hyperinflammatory response within the chronic wound bed leading to delayed healing. For example, research that analysed 120 DFUs of hospitalised patients for their microbiome composition, found 81.66% had an infection, and 23.3% of those had a polymicrobial infection.^60^ *S*. *aureus* presented as the largest coloniser (40.81%), followed by *Escherichia*. *coli* (34.69%) and *P*. *aeruginosa* (30.61%). Another study analysed 2963 patients with chronic wound samples in which 910 participants had DFUs, (other wound types included VLUs, non‐healing surgical wound and decubitus ulcer) *S*. *aureus* and *S. epidermidis* were the most frequent species identified in these samples.^61^ They also found bacterial composition was independent of chronic wound type, *S*. *epidermidis* was found to be more prevalent in DFUs, and *P*. *aeruginosa* exhibited at a higher percentage in chronic wounds which presented with biofilm formation. Moreover, anaerobes such as *Peptostreptococcus*, *Prevotella*, *Peptonipihlus*, *Fingelodia* and *Anaerococcus* have presented themselves as consistent members of the chronic wound microenvironment albeit at lower abundancies.^60^ Another study analysed the DFU microbiome (*n* = 52) using 16S rDNA amplicon sequencing and identified 13 phyla with *Proteobacteria*, *Firmicutes*, *Bacteroidetes*, *Actinobacteria* and *Fusobacteri*a as the most common isolates.^62^ *Staphylococcus* spp were identified in 49 of 52 samples suggesting that *Staphylococcus* spp are particularly pathogenic to DFUs. Phylogenetic analysis further determined the abundance of each species, identifying that 96.5% was *S*. *aureus* and 0.4% was *S. epidermis*. They also reported that the physical characteristics of wounds impacts microbial diversity. For example, deep ulcers presented with a more diverse microbiota with a higher abundancy of anaerobes and *Proteobacteria* whereas superficial ulcers presented with a higher abundancy of *Staphylococcus* spp.^63^, ^64^, ^65^, ^66^, ^67^, ^68^, ^69^, ^70^ These studies highlight the microbial complexity in chronic wounds and controlling the composition of polymicrobial community in chronic wound may be the key strategy to promote healing.

## 4 CURRENT TREATMENTS AGAINST CHRONIC INFECTIONS

A wide range of therapies and tools as part of a medical practitioner's armamentarium are available which aid in combating DFUs from varying angles (Table 1). The gold standard is surgical debridement of the wound bed, managing infection using antimicrobial agents, revascularisation procedures and the offloading of the wound.^71^ The ultimate goal for any wound is to achieve complete regeneration, albeit with scarring. and there are many novel ideas utilised in current research. For example, inhibiting matrixmetallaprotease‐9, a proteolytic enzyme responsible for degrading the extracellular matrix, was reported to accelerate wound healing in diabetic mice more effectively than the commonly used topical medicine, becaplermin, sourced from recombinant human derived growth factor which naturally aids in wound healing.^72^ From a microbiological perspective, wound dressings are usually employed to physically protect the wound from further contamination, or to treat an infected wound using dressings which incorporate antimicrobials. Wound dressings including alginate, hydrogels, hydrocolloids, foams and films all have their own unique benefits but also present with their own limitations, for example; poor biocompatibility and/or hydrophobicity.^73^ Smart dressings which incorporate polydimethysiloxanes seem to yield promising results due to their many benefits such as low cost, high biocompatibility and nontoxic properties.^74^ Dressings which include sensors that detect changes in pH due to bacterial load and trigger colour changes would effectively be easily identifiable by medical practitioners allowing rapid management strategies, known as ‘detect and manage’ smart dressings^75^ presenting as an exciting new way forward.

**TABLE 1:** An evaluation of the current treatment methods and dressings currently available to combat chronic wounds.

Treatment/Dressing/Method	Strengths	Weaknesses	Ideal for infected chronic wound	References
Gauze	Highly absorbent	Can cause wound trauma and pain upon removal	No but can halt minimal bacteria load in acute wounds	^125^, ^126^
	Inexpensive	Nonocclusive		
	Readily available	Bacteria can permeate		
Transparent films	Flexible	No control over exudate	Not to be used for infected wounds, provides an ideal moist environment for bacteria to proliferate	^126^, ^127^
	Permeable to water, oxygen and carbon dioxide			
	Impermeable to bacteria			
	Promote autolytic debridement			
Foam dressings	Allow gaseous exchange	Able to dry wound fast	Can be used to treat infection if changed daily	^128^, ^129^
	Can absorb wound drainage			
Hydrogels	High water content aids granulation	Exudate accumulation	Has been used to treat chronic wounds but not ideal to treat chronic infection	^130^, ^131^
	Easy application and removal	Maceration leads to bacterial proliferation		
	Non‐toxic	Low mechanical strength		
pH sensitive dressings	Can be incorporated into hydrogels	Suffers from high mechanical fragility	Yes, the incorporation in multiple dressings and a visible colour change in the presence of bacteria makes this an ideal and easy to use method for clinicians.	[136,137]
	Creates a conformational change when a change in pH is detected	Fouling by non‐specific absorption		
	Releases antimicrobials	Needs to be frequently calibrated		
Silver dressings	Traditional	Lack of evidence on pathogens which have invaded deep into the tissue	Yes, to treat superficial bacteria	^132^, ^133^
	Silver is broad spectrum antibiotic			
	Reduces inflammation in wound			
Alginate film with xylitol or gentamycin	Superior mechanical properties	Inhibit keratinocyte migration	Yes, the use of incorporated antimicrobials and the stimulation of macrophage migration makes this an ideal dressing	^132^
	Rheological properties			
	Effective against biofilm forming bacteria			
Microspheres	Can encapsulate multiple drugs at once	They may possibly migrate away from site of injection and cause organ damage	Yes but would need the use of a dressing to physically protect the wound from the environment	^132^
	Effectively reduce infection levels			

Antimicrobial agents are the most therapeutically, commercially used drugs. Insufficient doses or biofilms which block the effective penetration of antibiotics drive the evolution of persistent cells and ultimately lead to widespread multi‐drug resistance (MDR) in many microbes.^76^ This has become a festering problem for many decades with little progress in the development of new classes of antibiotics in the past 30 years, and MDR bacteria are causing high mortality rates.^77^, ^78^, ^79^ One of the most notable and famous examples of resistance is the widespread resistance to penicillin by *S*. *aureus* followed by resistance to methicillin (methicillin resistant *S*. *aureus* [MRSA]). Vancomycin is used as the ‘last line of defence’ against *S*. *aureus* infections however, during recent in vivo and ex vivo trials, vancomycin resistant *S*. *aureus* (VRSA) has already become an issue.^80^

Besides antibiotics, various methods to improve bactericidal strategies against infections have been employed. For example, the inhibitory effect of blue light treatment on *P*. *aeruginosa* virulence factors showed that a lethal dose of blue light inhibited staphylolysin, pseudolysin and pyocyanin virulence factors increasing antimicrobial susceptibility, which highlights a combinatorial approach with blue light therapy and antimicrobials.^81^ Silver nanoparticles have wide‐reaching applications including their use in regenerative materials, drug delivery and antimicrobials.^82^ Moreover, silver nanoparticles have been utilised in recent years for artificial transplantation, implantable medical devices, antibacterial vaccines and wound dressings.^83^ The antimicrobial efficacy of silver nanoparticles resides in their increased surface area and nanometric size, as well as their ability to efficiently disrupt the membrane, penetrate the bacterial membrane, and cause intracellular damage. Metal ion release, oxidative stress and non‐oxidative mechanisms also contribute to this antimicrobial effect.^84^ Novel methods include the utilization of common antibiotics; doxycyline and amoxicillin and their conversion to nanoform composites to acquire new pharmacological properties. The conversion to nanoscale drugs allowed the acquisition of new properties, such as wound healing and ulcer prevention and increased the antimicrobial penetration power against pathogenic microbes which infect wounds. However, MDR is always a growing issue, demanding novel techniques which do not promote MDR.^85^, ^86^, ^87^, ^88^

### 4.1 Novel treatment horizons: Phage therapy and microbiota transplantation

There has been a growing interest in exploring the potential of bacteriophages (phages) for the treatment of DFUs.^89^, ^90^, ^91^, ^92^, ^93^ Phage therapy represents an alternative therapeutic approach to antibiotics. Phages are obligate bacterial viruses that specifically lyse their host bacteria and disrupt bacteria biofilms by encoding enzymes such as lysins and depolymerases.^94^, ^95^, ^96^, ^97^ It has been determined that they are effective in reducing the microbial load in wounds and facilitating the expression of proteins that are associated with the process of wound healing. Phage ϕMR11, which encodes lysin MV‐L, can efficiently eliminate MRSA and VRSA in growing conditions.^98^ In an animal model, injecting the phage MR‐10 on the hind paw of diabetic mice infected with MRSA resulted in the resolution of the infections within a period of 5 days. This contrasts with the untreated mice, which required 15 days for the infections to be resolved.^99^ Apart from single phage, phage cocktail therapy combines different phages targeting diverse bacterial strains can efficiently kill a broad range of pathogen strains. AB‐SA01 cocktail is composed of three *S*. *aureus* Myoviridae phages. The application of AB‐SA01 cocktail for DFU treatment was tested on diabetic mice infected with MDR *S*. *aureus* isolates collected from DFU patients. The results exhibited a significant reduction in bacterial load, complying with a similar trend to the vancomycin‐treated group during the treatment period. AB‐SA01 treated mice had continued reduction of bacteria load after the cessation of treatment most likely due to phage self‐replication in their bacterial host, whereas vancomycin‐treated mice did not exhibit the same effect, highlighting an advantage of phages over antibiotic treatment.^89^ Additionally, unlike antibiotics which necessitate escalating concentrations that ultimately result in the ineffectiveness of MDR bacteria, phages possess the ability to selectively target MDR bacteria using relatively low initial doses.^92^ Of note, although the application of AB‐SA01 cocktail therapy in human clinical trials for DFUs has not yet been conducted, a human phase I trial has verified the safety and tolerability of AB‐SA01 therapy for chronic rhinosinusitis.^100^ Phages are bacterial viruses that infect bacterial hosts with a high specificity through bacterial host surface receptors. Interestingly, recent studies suggest that phage can also influence microbiome compositions, interact with eukaryotic cells, and modulate immune responses.^101^ Yet, the impact of phage therapy in treating chronic wound infection and modulating local immune responses to promote healing is under‐investigated.

Pairing phage cocktails with antibiotics is another possible therapeutic approach to improve treatment efficacy. An *S*. *aureus* biofilm was eliminated more efficiently by phage treatment along with antibiotic exposure afterwards.^102^ Diabetic mice with *S*. *aureus* infected hind paws exhibited significant reduction in bacterial load when subject to concurrent treatment which phage therapy and linezolid.^99^ Similarly, the combination of ciprofloxacin and phage has demonstrated better efficiency in inhibiting the growth of *P*. *aeruginosa*, compared with bacteria growth inhibited by single phage or ciprofloxacin separately.^103^ The effect of treating *P*. *aeruginosa* biofilm infection with antibiotics was improved when infection was exposed to phages.^104^ Phages have emerged as a potential therapeutic therapy for the treatment of bacterial infection. However, the effectiveness of using phage treatment for polymicrobial infections is limited due to their specificity of bacterial hosts. Moreover, the interaction between phages and bacteria can result in the quick emergence of bacterial resistance to the phage, although the phage has continual adaptation in response to bacterial changes.^105^, ^106^, ^107^ Cocktails broaden the range of phage applications through the amalgamation of multiple phages and reduce the generation rate of phage‐resistant strains compared with single phage.^93^ Due to various limitations such as the horizontal evolution of bacteria, the limited host range of bacteriophages, the removal of endotoxins in preparations, the technical feasibility of isolation, the mode of administration, rapid clearance, and immune rejection, there is a lack of clinical trials on the application of phage therapy for DFUs. Notwithstanding these limitations, the aforementioned investigations employing a blend of cocktails with antibiotics in mice models of diabetes have been showcased, providing support for a potentially effective therapeutic intervention.

Studies investigating the microbial composition of DFUs have found they predominately consist of facultative and strict anaerobes.^108^, ^109^ Moreover, the presence of strict anaerobes has been linked with reoccurring and persistent infections.^110^, ^111^, ^112^ Interestingly, chronic wounds that display a prolonged healing time exhibit the least microbiome diversity.^112^ This phenomenon can be attributed to empiric antimicrobial treatments utilised at the initial stages of hospitalisation and the acquisition of highly virulent and resistant opportunistic pathogens.^113^ For example, *P*. *aeruginosa* and *S*. *aureus* co‐infections are more virulent than single infections as both bacteria are trying to outcompete each other for the same nutrient availability.^108^ This leads to the suppression of other bacterial species present in the polymicrobial community and to spatial reorganisation within the biofilm. *P*. *aeruginosa* is normally found deeper in more hypoxic regions while *S*. *aureus* is located closer to the surface.^114^ This can be attributed to the faster growth rate, motility and metabolic adaptability of *P*. *aeruginosa* in a hypoxic environment, allowing the bacterium to supress *S*. *aureus* colonisation in the deeper regions of the wound bed. In patients with no comorbidities the skin microbiome can be beneficial in eliciting a localised immune reaction that in turn induces a systemic immune response.^115^ Hence, skin commensals are kept in equilibrium with the skin barrier and are unable to further colonise the wound. However, in chronic wounds this equilibrium is disrupted because of the underlying systemic disease of the patient, making it difficult to treat with commensal probiotics alone. Studies have shown the importance of maintaining healthy homoeostasis at sites of infection via microbiome transplantation, with the primary focus being on faecal transplantation for the treatment of *Clostridium difficile* infections and for surgical operations such as colostomy and ileostomy.^116^, ^117^, ^118^, ^119^, ^120^ However, microbiota transplantation remains an underdeveloped area of research for the treatment of chronic wounds. It is generally accepted that a diverse microbiome is considered the "right recipe". However, the exact number and type are highly debated, and can vary widely in different skin locations in different individuals.

In acute wounds *Staphylococcal* (*S*. *epidermidis*, *S. warneri*, *S. hemolyticus*) and *Micrococcal* (*M*. *luteus*) species can migrate to the site of injury and promote skin barrier restoration.^121^, ^122^ Specifically, *S*. *epidermidis* promotes wound healing via TLR‐2 signalling and the production of lipoteichoic acid, which helps reduce inflammation and promotes transition into the proliferation phase.^123^ For many years *S*. *epidermidis* has been believed to be a harmless skin commensal that antagonises *S*. *aureus* and aids in acute wound healing.^117^ However, recent genomic and epidemiological studies have shown that nosocomial lineages have emerged harbouring virulence factors that aid in invasion. These newly acquired pathogenicity factors can range from biofilm formation, cell toxicity, methicillin resistance and post infection induction of pro‐inflammatory biomarkers (IL‐8, IL‐6, TNF‐a) correspondent to *Staphylococcal* sepsis.^110^ Interestingly, a recent murine study highlighted the significance between *S*. *aureus* and inducing an immune response (IL‐1b/IL‐1 R/MyD88) in relation with accelerated wound healing in acute wounds.^121^ Additionally, a study investigating microbiome composition in chronic wounds demonstrated a direct correlation of *S*. *aureus* strain specificity with disease severity and localised immune response.^124^ This further supports the complex interactions between skin microbiota composition, host immunity and wound healing progression. However, chronic wounds such as DFUs display an impaired immune response because of a reduced blood supply to the extremities.^122^ This leads to the formation of a hypoxic and nutrient rich microenvironment in which bacterial infections can proliferate and form polymicrobial biofilms.^113^

DFUs and most chronic wounds show non‐uniformed characteristics among patients and relying on a ‘one‐size‐fits‐all’ therapy can limit the success of regeneration for each patient. Phage targeted therapies and microbiome transplants should be researched more as a multi‐combinatorial treatment strategy with the latter providing a diverse microbiota, to limit the spread of pathogens and the formers targeting any pathogens with the potential to proliferate, thereby reducing microbial pressure at the wound site, which allow the immune system to mount an effective defence. There are many conflicting views within the literature of whether a wound bed needs to be sterile or host a diverse microbiota for optimal healing.^62^, ^64^ Maintaining sterile wound environment is the main and only purpose for developing antimicrobial wound dressing materials. For a sterile environment, any microorganism would need to be eliminated for the duration of the wound regeneration cascade. This is of course not feasible, with research suggesting a diverse microbiome needs to be present to halt the over‐proliferation of pathogenic, biofilm forming micro‐organisms.^64^ This supresses the secretion of virulence factors by these microbes, which halt healing and allows the immune system to mount an effective offence, eliminate microbes, both healthy and pathogenic along with the gradual closure of the wound. Moreover, a wound environment underwent antibiotic treatment to eliminate existing healthy and pathogenic commensals provides an opportunity for new pathogenic bacteria without the competition from commensal microbiota to overpopulate the wound bed and further hyper‐perpetuate the late inflammatory stage of wounding. The use of antibiotics is becoming more controversial with each passing year, since the widespread and rapid development of resistance poses a huge problem of treating infection effectively. Clearly alternative and novel methods are required, and phage targeted therapy and microbiota transplantation may be promising.

## 5 CONCLUSIONS

Regardless of all the modern therapies and personalised medicine, chronic, non‐healing wounds remain a silent pandemic. The bioburden of the chronic wound microenvironment of the DFU frequently results in amputation or even death. The aetiology of the underlying neuropathy and the combination of polymicrobial biofilms provide microorganisms with multiple layers of defence to thrive and proliferate in the nutrient rich DFU. Conventional technologies for example, standard culturing techniques and DNA sequencing have limitation when accurately identifying chronic wound isolates and their exact abundancies, therefore most research will either under or over report the prevalence of microbes. Current technologies are advancing at a rapid rate and perspectives which combine the molecular, cellular, physiological and microbiological therapies should be commonplace going forward. A relationship can be distinguished between the landscape of chronic wound microbes and the metabolic, proteomic, metagenomic and transcriptomic profiles. Moving forward the use of these approaches could accurately identify species and abundancies and perhaps give an indication to the early stages of wound infection before the formation of established chronic biofilms. Moreover, these approaches used to identify pathogenic factors presented within the chronic wound micro‐environment could effectively be utilised as biomarkers of certain pathogens present, thereby probing the counterselection of pathogenic bacteria by the targeted phage therapy to inhibit pathogens and contribute to a healthy cutaneous microbiota, releasing pressure from the wound bed and allowing the immune system to mount an effective defence. Antimicrobial agents would still be required, and could potentially be combined with phage/microbiome transplants to minimise the emergence of resistance profiles. The research into the combination of phage and microbiome transplants is still in its infancy, but success of these therapies in gastrointestinal cases gives hope that this might be translated into a cutaneous/infected wound healing environment.

## CONFLICT OF INTEREST STATEMENT

None to declare.

## AUTHOR CONTRIBUTIONS

**Paul Norton**: Writing – original draft (lead); Writing – review & editing (equal). **Pavlos Trus**: Writing – original draft (supporting); Writing – review & editing (supporting). **Fengyi Wang**: Writing – original draft (supporting); Writing – review & editing (supporting). **M. Julie Thornton**: Conceptualization (equal); Resources (equal); Supervision (equal); Writing – original draft (supporting); Writing – review & editing (equal). **Chien‐Yi Chang**: Conceptualization (equal); Funding acquisition (lead); Resources (equal); Supervision (equal); Writing – original draft (supporting); Writing – review & editing (equal).

## ETHICS STATEMENT

Not applicable.

## Data Availability

All data and materials are published in the cited references.
